# Left Atrial Reservoir Strain in Cardiovascular and Systemic Disease: Advances and Clinical Applications From Physiology to Practice

**DOI:** 10.31083/RCM46198

**Published:** 2025-12-25

**Authors:** Andrea Sonaglioni, Gian Luigi Nicolosi

**Affiliations:** ^1^Division of Cardiology, IRCCS MultiMedica, 20123 Milan, Italy; ^2^Division of Cardiology, Policlinico San Giorgio, 33170 Pordenone, Italy

**Keywords:** left atrial function, left atrial reservoir strain, physiology, clinical applications, limitations

## Abstract

Traditional parameters, such as left atrial size or volume, typically reflect chronic pressure and volume overload; however, these abnormalities only become evident at advanced stages, often missing early signs of dysfunction. In contrast, left atrial reservoir strain (LASr), measured by speckle-tracking echocardiography, offers a sensitive and dynamic assessment of atrial mechanics, integrating atrial compliance with left ventricular diastolic interaction. Moreover, impaired LASr reflects atrial stiffness and fibrosis, and correlates with elevated filling pressures, making the LASr parameter a comprehensive biomarker of left-sided cardiac function. Indeed, LASr has demonstrated diagnostic and prognostic value across a wide spectrum of conditions. In heart failure with preserved ejection fraction, LASr refines the assessment of diastolic dysfunction and predicts hospitalization and mortality. In atrial fibrillation, reduced strain correlates with atrial fibrosis and left atrial appendage dysfunction, identifying patients at increased risk of arrhythmia recurrence and thromboembolism. In valvular disease, LASr uncovers subclinical remodeling and stratifies risk even in patients with apparently moderate aortic stenosis. Meanwhile, in addition to cardiovascular disease, LASr can detect early atrial impairment in systemic disorders such as hypertension, diabetes, obesity, and amyloidosis, often before structural enlargement becomes evident. Our group has shown that LASr predicts persistent hypertension after gestational hypertensive disorders, reveals subclinical diastolic dysfunction in idiopathic pulmonary fibrosis, non-invasively predicts left atrial appendage thrombus in atrial fibrillation, stratifies outcomes in moderate aortic stenosis, and provides prognostic information in acute ischemic stroke. This narrative review outlines the physiological basis, technical considerations, and clinical applications of LASr, discusses its limitations and future perspectives—including multimodality imaging and artificial intelligence—and underscores its transition from a research metric to a dynamic biomarker ready for clinical practice.

## 1. Introduction

The left atrium (LA), once regarded as a passive chamber, is now recognized as a dynamic contributor to global cardiac performance. It fulfills three essential functions—reservoir, conduit, and booster pump—that modulate ventricular filling and systemic hemodynamics. Traditionally, LA assessment relied on morphologic indices such as diameter, area, and volume. These volumetric measures correlate with chronic pressure and volume overload, and LA enlargement has long been established as a predictor of atrial fibrillation (AF), stroke, and heart failure (HF) [1]. Yet, enlargement represents a late stage of remodeling. Subclinical functional impairment may precede dilation by years, meaning that reliance on size alone underestimates early atrial dysfunction and delays risk stratification. Traditional echocardiographic indices, while useful, therefore fail to capture the dynamic mechanical behavior of the atrium. This limitation has driven the search for parameters capable of identifying early atrial dysfunction before irreversible structural remodeling occurs.

The advent of two-dimensional speckle-tracking echocardiography (2D-STE) revolutionized atrial imaging by enabling quantification of atrial mechanics. Among the derived indices, left atrial reservoir strain (LASr)—the peak positive longitudinal strain during ventricular systole—has emerged as the most reproducible and clinically relevant marker. LASr integrates myocardial properties and ventricular-atrial interaction: it reflects atrial compliance and fibrosis burden [2, 3] and captures the influence of left ventricular relaxation and filling pressures [4, 5]. By doing so, LASr provides a comprehensive, noninvasive biomarker of atrial health that surpasses volumetric surrogates.

From a physiological perspective, LASr quantifies the elastic expansion of the atrial wall during ventricular systole, directly mirroring atrial compliance and left ventricular diastolic function. Consequently, it serves as a sensitive index of atrial–ventricular coupling and myocardial stiffness, linking functional assessment to underlying structural changes.

Clinical studies have demonstrated the value of LASr in diverse cardiovascular conditions. In HF with preserved ejection fraction (HFpEF), LASr improves diagnostic accuracy and prognostic assessment, identifying patients at higher risk of hospitalization and mortality [6, 7]. In AF, LASr correlates closely with atrial fibrosis, left atrial appendage (LAA) dysfunction, and thromboembolic risk, thereby predicting recurrence after ablation and risk of stroke [8, 9]. In valvular disease, LASr reveals early atrial involvement in moderate aortic stenosis [10] and in moderate mitral regurgitation [11], stratifying asymptomatic patients into prognostic groups and predicting adverse outcomes. Furthermore, LASr abnormalities anticipate atrial cardiomyopathy in systemic disorders such as hypertension [12], diabetes [13], obesity [14], and amyloidosis [15]. Together, these findings position LASr as a sensitive and integrative biomarker that bridges myocardial mechanics, hemodynamics, and clinical outcomes—extending its utility beyond traditional echocardiographic measurements.

Recent reviews reinforce these findings. O’Neill *et al*. [16] and Rusali *et al*. [17] summarized LASr as an earlier and more sensitive marker of pathology than LA volume, often altered years before geometric remodeling occurs. Similarly, Cau *et al*. [18] demonstrated that cardiac magnetic resonance (CMR) feature tracking provides reproducible atrial strain assessment, although echocardiography remains the most accessible modality. Kupczyńska *et al*. [19] emphasized the need for methodological standardization, while Wang *et al*. [20] introduced semi-automated algorithms that may facilitate LASr integration into routine workflows.

Our group has extended these insights to unconventional contexts. We demonstrated that impaired LASr during pregnancy predicts persistent hypertension after gestational hypertensive disorders [21, 22] and that patients with idiopathic pulmonary fibrosis (IPF) exhibit early atrial dysfunction despite preserved left ventricular (LV) systolic function [23]. Transthoracic LASr can predict LAA thrombus, potentially reducing the need for invasive transesophageal echocardiography (TEE) in selected AF patients [24, 25] and identifies high-risk subgroups in moderate aortic stenosis [26]. LASr measured acutely in ischemic stroke patients predicts short-term outcomes even in sinus rhythm [27]. These findings underscore LASr’s prognostic and diagnostic robustness across a spectrum of diseases.

Overall, this body of evidence underscores a paradigm shift: the left atrium should no longer be evaluated solely by size but also by its functional mechanics. LASr captures early, reversible stages of dysfunction and offers an accessible parameter that connects imaging physiology with clinical outcomes.

The present review therefore aims to: (1) outline the physiological basis of LASr and its determinants; (2) discuss technical considerations and current standardization efforts; (3) summarize its clinical applications across cardiovascular and systemic diseases; and (4) address limitations and future perspectives to support its translation from research into everyday clinical practice.

## 2. Physiology, Measurement, Technical Aspects and Clinical Challenges of LASr

The left atrium has long been described as the “forgotten chamber”, overshadowed by the more dynamic left ventricle. Yet careful physiological studies revealed decades ago that the atrium is not a passive conduit but a remarkably adaptive chamber, one that changes its role within every heartbeat. Its three phasic functions—reservoir, conduit, and booster pump—summarize a complex interplay of myocardial deformation, ventricular relaxation, and pulmonary venous return [28]. During ventricular systole and isovolumic relaxation, the atrium stretches as a reservoir, storing blood from the pulmonary veins. Once the mitral valve opens, it becomes a conduit, allowing passive flow into the ventricle. Finally, in late diastole, it contracts, delivering a booster pump contribution that can account for up to 30% of ventricular filling in older individuals or in those with stiff ventricles [29]. These three phasic components are highly interdependent and dynamically modulated by changes in loading conditions, atrial compliance, and ventricular diastolic properties, reflecting the continuous mechanical coupling between both chambers.

From a physiological standpoint, the reservoir phase represents the elastic expansion of the atrial wall during ventricular systole and depends mainly on left atrial compliance, left ventricular longitudinal shortening, and the efficiency of pulmonary venous return. The conduit phase, which facilitates passive ventricular filling in early diastole, is influenced by ventricular relaxation and suction, whereas the booster pump phase depends on atrial contractility and synchronized atrioventricular activation. As diastolic dysfunction develops, conduit function declines first, the booster phase compensates, and progressive stiffening of the atrial wall ultimately reduces reservoir function. Consequently, reservoir impairment becomes a key marker of advanced atrial–ventricular uncoupling and increased filling pressures.

What was missing for many years was a sensitive, non-invasive measure that could capture atrial mechanics early in this sequence. Strain imaging can fill that gap. By tracking the deformation of the atrial wall, clinicians could quantify how well the atrium stretches, a measure closely tied to both myocardial compliance and ventricular diastolic load. The peak positive longitudinal strain achieved during ventricular systole, termed LASr, emerged as the most robust and reproducible marker. LASr declines with atrial fibrosis—demonstrated both on histology [30] and on CMR with late gadolinium enhancement [2]—and correlates strongly with invasive measures of LV filling pressures [31, 32]. Thus, LASr represents a composite index that integrates three major determinants: intrinsic atrial myocardial compliance and fibrosis burden, left ventricular systolic and diastolic performance, and overall atrioventricular coupling. This dual sensitivity to structural and hemodynamic factors explains why LASr encapsulates multiple dimensions of cardiovascular dysfunction within a single parameter.

For decades, echocardiographers relied on surrogate measures. Left atrial diameter and later the left atrial volume index (LAVi) became standard, endorsed by guidelines as a marker of chronic diastolic burden [33]. Doppler indices such as transmitral inflow or pulmonary venous flow were also widely used. Yet all of these have limitations: size changes only slowly, Doppler patterns are highly load-dependent, and both fail in AF when the atrial contraction disappears [34]. LASr, by contrast, directly interrogates myocardial deformation and has proved more sensitive to early dysfunction [35]. Its ability to detect subtle mechanical impairment before structural remodeling or volumetric enlargement occurs makes it a pivotal parameter for identifying early, potentially reversible stages of atrial dysfunction.

**Fig. 1.**
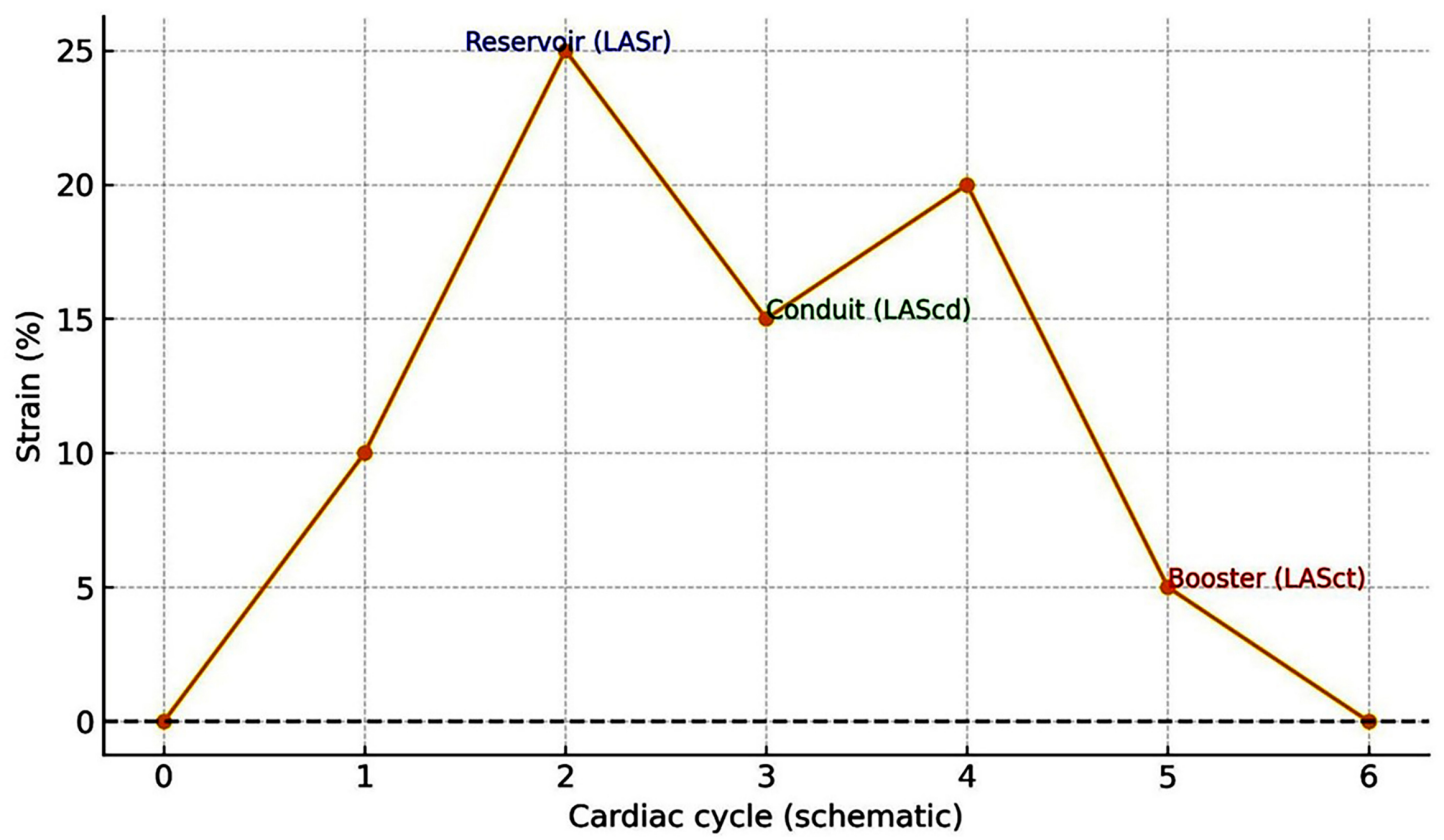
**Left atrial phasic strain curve**. The curve illustrates the three functional phases of left atrial deformation—reservoir, conduit, and booster (contractile)—as derived from speckle-tracking echocardiography or other imaging modalities. During the reservoir phase (ventricular systole), the atrium fills with pulmonary venous return, and the peak positive longitudinal strain is recorded as LASr. In the conduit phase (early diastole), LAScd reflects passive emptying of the atrium into the ventricle, while in the booster phase (late diastole), LASct represents active atrial contraction that completes ventricular filling. This schematic depiction illustrates the universal strain pattern common to 2D/3D echocardiography, cardiac magnetic resonance, and computed tomography, and is not modality-specific. LASr, left atrial reservoir strain; LAScd, left atrial conduit strain; LASct, left atrial contractile strain.

Fig. 1 schematically illustrates the three phasic components of left atrial strain—reservoir, conduit, and booster—within the cardiac cycle, emphasizing that LASr corresponds to the peak strain reached during the atrial filling (reservoir) phase.

The technique of measuring LASr is simple in principle but requires rigor in execution. Apical four- and two-chamber views should be acquired without foreshortening, as even slight angulation can inflate strain values. The endocardial border is traced carefully, excluding pulmonary veins and the appendage. Frame rates above 60 fps are optimal, and most laboratories now use the R-wave of the electrocardiogram (ECG) as the zero reference for strain curves. Although some advocate the P-wave method in sinus rhythm to align with atrial contraction, consensus favors R-wave gating for its consistency across rhythms [36]. Accurate strain analysis also requires optimal image quality, steady heart rate, and minimal translational motion, as noise or tracking loss can markedly alter calculated values. Early work often used left ventricular strain software, but dedicated atrial modules have since improved accuracy. Even so, inter-vendor variability persists, with systematic absolute differences of 2–4% that remain unresolved and significantly affect the definition of normal cut-off values, despite international standardization efforts [37]. Accordingly, using the same ultrasound platform and software during serial evaluations is strongly recommended to ensure longitudinal comparability. Ongoing standardization initiatives from the EACVI/ASE/Industry Task Force [36] have provided unified recommendations on acquisition, tracking methodology, and reporting formats. These initiatives represent an essential step toward the development of vendor-independent software and universally accepted reference ranges that will facilitate the inclusion of LASr in future clinical guidelines.

Beyond two-dimensional speckle tracking, other modalities enrich the picture. Three-dimensional speckle tracking eliminates out-of-plane motion and offers comprehensive atrial tracking. Normal values are however lower with 3D imaging, typically 25–30% compared with 39–45% for 2D [38], likely due to algorithmic differences and the reduced spatial resolution and frame rate inherent to 3D techniques. CMR feature tracking provides high spatial resolution and directly correlates reduced LASr with the extent of atrial fibrosis, particularly in AF cohorts [2, 39]. Computed tomography (CT) feature tracking has more recently been validated, showing good agreement with echocardiography [40, 41, 42], although radiation and contrast exposure limit routine use. The consistency of results across imaging platforms confirms that LASr is not an artifact of a single technology but rather a reproducible physiological signal that reflects atrial compliance and left ventricular filling pressures.

Normal values vary with age, sex, and modality. In healthy adults studied by 2D-STE, LASr typically ranges between 39–45% [43]. Values decline steadily with aging, particularly in men [44]. Children and adolescents exhibit higher strain, approaching 50%, necessitating pediatric-specific charts [45]. With 3D-STE, values are systematically lower [38], while CMR values tend to fall between those of 2D and 3D, correlating closely with fibrosis [39]. The lowest LASr values are observed with CT feature tracking [40, 41, 42], likely due to its lower frame rate. These differences highlight the need for population- and modality-specific reference ranges, as well as caution when interpreting results across imaging techniques.

Table 1 (Ref. [2, 38, 39, 40, 41, 42, 43, 45]) summarizes reference values across populations and modalities. All these biological, technical, and methodological sources of variability represent major challenges for clinical interpretation and underline the importance of contextual assessment in each individual patient.

**Table 1.** **Normal reference values for LASr across modalities and populations**.

Population/Modality	Typical LASr (%)	LASr magnitude
Healthy adults (2D-STE) [43]	~39–45	Declines with age
Children/adolescents (2D-STE) [45]	~45–50	Higher than adults
3D-STE [38]	~25–30	Systematically lower vs. 2D
CMR feature tracking [2, 39]	~30–35	Between those of 2D and 3D
CT feature tracking [40, 41, 42]	~19–25	Lower than 2D-STE

Note: 2D-STE, two-dimensional speckle tracking echocardiography; 3D-STE, three-dimensional speckle tracking echocardiography; CMR, cardiac magnetic resonance; CT, computed tomography; LASr, left atrial reservoir strain.

This interpretative challenge is also increased by the fact that LASr is sensitive to loading conditions. Tilt-table studies and controlled preload reduction confirm that LASr falls when venous return decreases, though less so than atrial volumes [46]. Clinicians should therefore interpret LASr values within the hemodynamic and clinical context, particularly in conditions characterized by rapid changes in preload, blood pressure, or intrathoracic pressure. Rhythm introduces complexity: reservoir strain remains interpretable in AF, but conduit and booster phases are unreliable. Multi-beat averaging is therefore recommended, and pragmatic three-beat protocols have demonstrated predictive power for stroke beyond CHA_2_DS_2_-VASc [47]. Feasibility is generally high, exceeding 90% in routine practice, but certain populations—those with obesity, lung disease, or postsurgical chest anatomy—may pose difficulties. In these cases, CMR or CT can provide alternatives [40, 48].

Our own investigations have extended LASr into challenging contexts, underscoring its robustness. In pregnancy, we showed that LASr can be reliably acquired despite hyperdynamic physiology and that its impairment predicts persistent hypertension postpartum [21, 22]. In AF, we demonstrated that transthoracic LASr offers a non-invasive means of predicting left atrial appendage thrombus and serves as a safer substitute for TEE in situations where the latter is impractical, such as during infectious disease outbreaks [24, 25]. In acute ischemic stroke, LASr proved both feasible and prognostically informative even in emergency settings [27]. These experiences confirm that LASr is not confined to ideal laboratory conditions but can be integrated into real-world clinical practice.

In sum, LASr bridges physiology and practice. It condenses the complex interplay of compliance, fibrosis, and filling pressures into a single dynamic parameter. By tracing the atrium’s stretch, clinicians can detect dysfunction earlier than with volume or Doppler, assess risk more accurately across modalities, and apply the measure in diverse and even urgent clinical contexts. Nevertheless, biological variability, vendor differences, and hemodynamic influences remain important limitations that should be carefully considered when interpreting individual LASr values in daily clinical decision-making.

## 3. LASr in Heart Failure and Atrial Fibrillation 

The clinical syndromes of heart failure and AF remain the two most common arenas in which LASr has been tested, validated, and applied. Both conditions are deeply intertwined: the stiffened ventricle of heart failure burdens the atrium, while AF is both a cause and a consequence of atrial myopathy. The principal value of LASr in these contexts lies in its ability to capture the cumulative effect of atrial compliance, ventricular diastolic load, and atrioventricular coupling in a single, reproducible metric.

HFpEF is perhaps where LASr has found its most distinctive role. Diagnosing HFpEF is notoriously difficult because patients present with nonspecific symptoms, preserved left ventricular ejection fraction (LVEF), and often equivocal diastolic indices. Guideline algorithms rely on Doppler indices (E/e^′^, tricuspid regurgitation velocity), atrial size, and natriuretic peptides, but many patients fall into an “indeterminate” category. Invasive hemodynamics remain the gold standard but are impractical in routine care. LASr has emerged as a sensitive, non-invasive marker of elevated LV filling pressures and diastolic dysfunction. Large multicenter studies demonstrated that a LASr <23–25% is strongly associated with invasively confirmed HFpEF [32]. Moreover, impaired LASr predicted hospitalization and mortality independent of conventional parameters [49]. Clinically, incorporating LASr into diastolic function algorithms reduces the proportion of patients classified as “indeterminate”, allowing earlier and more confident diagnosis.

In heart failure with reduced ejection fraction (HFrEF), the prognostic significance of LASr is particularly compelling. While LVEF and dimensions/volumes remain the primary metrics guiding management, LASr brings added value by reflecting atrial contributions to diastolic filling and chronic remodeling. In cohorts of patients with dilated cardiomyopathy (DCM), CMR feature tracking–derived LASr has shown independent predictive power for adverse outcomes, including all-cause mortality, heart failure hospitalization, implantable cardioverter defibrillator (ICD) implantation, and heart transplantation—even after adjustment for conventional LV systolic indices [50, 51]. Thus, LASr complements LVEF by providing an integrative measure of atrial–ventricular compliance that mirrors chronic diastolic burden.

Similarly, LASr correlates with exercise capacity, underlining the physiological significance of atrial reservoir function in functional limitation. In patients with heart failure (both HFrEF and HFpEF), lower LASr correlates linearly with reduced peak oxygen consumption (VO_2_) and skeletal muscle endurance, suggesting utility as a marker of integrated cardiac and peripheral exercise limitation [52]. This relationship underscores the role of the atrium as a determinant of forward stroke volume during exertion.

Even in Takotsubo syndrome, characterized by acute LV stunning followed by recovery, LA reservoir and booster pump strain are acutely impaired during the subacute phase and begin to normalize during convalescence—highlighting the dynamic atrioventricular coupling in this condition [53]. Such findings emphasize that LASr is not merely a marker of chronic remodeling but also a sensitive indicator of transient hemodynamic stress and recovery.

**Fig. 2.**
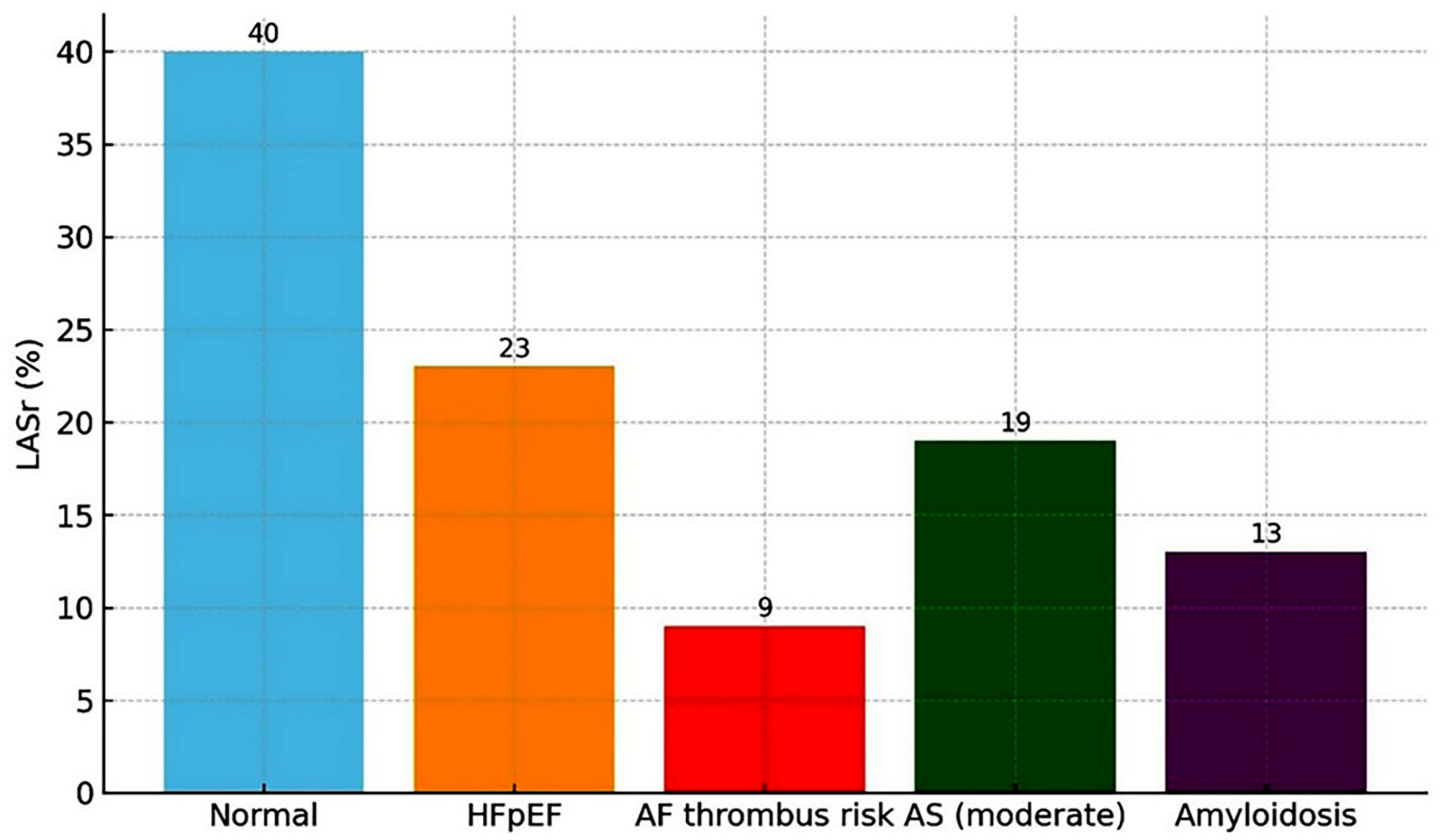
**Bar chart showing 2D-STE–derived LASr thresholds across health and disease states**. Normal values are highest (~40%), while progressively reduced thresholds are observed in HFpEF, atrial fibrillation with thrombus risk, moderate aortic stenosis, and cardiac amyloidosis, highlighting the role of LASr in diastolic function assessment and risk stratification. 2D, two-dimensional; AF, atrial fibrillation; AS, aortic stenosis; HFpEF, heart failure with preserved ejection fraction; LASr, left atrial reservoir strain; STE, speckle tracking echocardiography.

What makes LASr particularly attractive to clinicians is its role in simplifying diastolic dysfunction assessment. Traditional diagnostic algorithms often yield indeterminate results despite being multi-step and complex. When LASr is incorporated, diagnostic agreement with invasive measures improves and the “indeterminate” category decreases significantly—making clinical evaluation more decisive and efficient and increasing concordance with invasive measures [54]. Accordingly, current data support the integration of LASr as a quantitative, physiology-based complement to Doppler parameters in the assessment of LV diastolic function. Fig. 2 shows a schematic illustration of how LASr contributes to diastolic assessment and risk stratification in heart failure and AF.

The relevance of LASr extends beyond diagnosis to prognosis. In HFpEF, LASr predicts hospitalization and mortality even after adjusting for natriuretic peptides, LV mass, and systolic function [55, 56, 57]. In HFrEF, it independently forecasts outcomes across etiologies, including ischemic and non-ischemic dilated cardiomyopathy [58, 59, 60]. Importantly, LASr stratifies risk independently of LVEF, highlighting its complementary role: whereas ejection fraction reflects systolic performance, LASr reveals the integrated diastolic load and atrial compliance that drive prognosis.

Atrial fibrillation is both a cause and a consequence of atrial myopathy. Reservoir strain, by capturing atrial compliance, mirrors the structural substrate that sustains AF. Patients with persistent AF show markedly lower LASr than those with paroxysmal AF, and lower LASr predicts difficulty maintaining sinus rhythm after cardioversion or ablation [61, 62]. Importantly, LASr correlates with the extent of atrial fibrosis on CMR, confirming its biological plausibility as a surrogate of the arrhythmogenic substrate [2]. The practical application is clear in AF ablation: LASr provides a non-invasive estimate of the structural remodeling that determines rhythm-control success. Several studies have shown that patients with LASr below ~18–20% have higher recurrence rates after ablation [63, 64]. Conversely, those with preserved LASr are more likely to remain in sinus rhythm. This metric can thus assist clinicians in patient selection and counseling prior to ablation procedures.

Perhaps the most clinically actionable insight relates to thromboembolism. The LAA is the main site of thrombus formation in AF, and its function parallels atrial reservoir mechanics. Reduced LASr predicts LAA thrombus and spontaneous echo contrast even in the absence of TEE [24, 25]. Moreover, LASr adds to risk prediction beyond CHA_2_DS_2_-VASc score. Large observational cohorts showed that patients with LASr <20% carried a higher risk of stroke, even after adjusting for conventional risk scores [65]. Therefore, LASr may represent a novel imaging biomarker for thromboembolic risk stratification, refining anticoagulation decisions and identifying at-risk patients who might benefit from intensified surveillance. Our study group has confirmed these observations by showing that transthoracic LASr can non-invasively predict LAA thrombus, possibly eliminating the need for the invasive TEE examination [24, 25]. We also confirmed that LASr measurement is feasible and prognostically informative in the acute stroke setting, underscoring its clinical utility when decisions must be made rapidly [27].

In summary, LASr bridges diagnostic and prognostic domains in both HF and AF, providing a unifying metric that links atrial structure, ventricular load, and systemic hemodynamics.

Table 2 (Ref. [24, 25, 27, 53, 55, 56, 57, 58, 59, 60, 63, 64]) summarizes 2D-STE–derived LASr thresholds and prognostic value in HF and AF.

**Table 2.** **2D-STE–derived LASr thresholds and prognostic value in HF and AF**.

Clinical setting	LASr cut-off (%)	Clinical implication
HFpEF diagnosis [55, 56, 57]	<23–25	Identifies elevated filling pressures
HFrEF prognosis [58, 59, 60]	<20	Predicts hospitalization/mortality
Takotsubo syndrome [53]	~15	Reflects acute dysfunction, improves with recovery
AF ablation [63, 64]	<18–20	Predicts AF recurrence
AF thrombus risk [24, 25]	<10–12	Predicts LAA thrombus/SEC
AF stroke risk [27]	<20	Predicts stroke beyond CHA_2_DS_2_-VASc

Note: 2D-STE–derived LASr thresholds and prognostic value in HF and AF. AF, atrial fibrillation; CHA_2_DS_2_-VASc, Congestive heart failure, Hypertension, Age ≥75 (doubled), Diabetes, Stroke/TIA (doubled), Vascular disease, Age 65–74, Sex category (female); HF, heart failure; HFpEF, heart failure with preserved ejection fraction; HFrEF, heart failure with reduced ejection fraction; LAA, left atrial appendage; LASr, left atrial reservoir strain; SEC, spontaneous echo contrast.

## 4. LASr in Valvular Disease, Stroke, and Systemic Disorders

The study of valvular disease provides perhaps the most compelling evidence that the atrium is more than a passive bystander. Aortic stenosis, mitral regurgitation, and mitral stenosis each burden the atrium in distinct ways, and LASr captures these burdens well before structural enlargement occurs. By quantifying the mechanical consequences of chronic pressure or volume overload, LASr complements conventional valve parameters and reveals early atrial–ventricular uncoupling.

In aortic stenosis, the stiff ventricle transmits pressure backwards, raising left atrial afterload. Conventional severity grading relies on valve area and gradients, yet these do not reflect the state of atrial–ventricular coupling. Multiple studies have shown that LASr declines in patients with moderate aortic stenosis, even when volumes remain normal, and that reduced LASr identifies individuals at higher risk of adverse events [26, 66]. After valve replacement or transcatheter aortic valve implantation (TAVI), LASr improves in parallel with LV unloading, and recovery of strain predicts better symptomatic outcomes [67]. Thus, LASr acts not only as a marker of chronic remodeling but also as a dynamic indicator of reverse atrial adaptation following hemodynamic correction, providing a functional readout of therapeutic benefit.

Mitral regurgitation (MR) provides a different paradigm. The atrium is subject to chronic volume overload, often leading to massive enlargement. Yet enlargement alone does not tell the full story. LASr can distinguish between patients who remain compensated and those who are beginning to decompensate. Preserved LASr in MR suggests the atrium is still compliant and able to buffer regurgitant flow, whereas reduced LASr signals loss of compliance, pulmonary venous hypertension, and worse surgical outcomes [68]. Accordingly, LASr may refine the timing of surgical or transcatheter intervention, identifying patients at risk of postoperative atrial dysfunction even before overt symptom onset.

In mitral stenosis (MS), the narrowing of the valve creates a pressure gradient across the atrium. Reservoir strain declines proportionally with severity, reflecting not only the mechanical obstruction but also the chronic atrial remodeling induced by elevated pressures [69]. Even after successful percutaneous mitral valvuloplasty, improvement in LASr can track the hemodynamic relief achieved. This behavior highlights LASr as an integrative functional parameter that reflects both the direct valvular burden and the secondary atrial response to chronic pressure elevation.

Beyond the valve clinic, LASr has assumed a central role in the debate about atrial cardiomyopathy and stroke. While AF is the best-known arrhythmic risk factor for embolism, accumulating evidence shows that atrial dysfunction itself predisposes to thrombogenesis. Reduced LASr has emerged as one of the strongest echocardiographic predictors of stroke risk, even in individuals without documented AF. In population studies, people with low LASr but in sinus rhythm carried a higher risk of ischemic stroke, suggesting that impaired reservoir function is part of a broader atrial myopathy that fosters thrombosis [70]. This observation expands the concept of “embolic stroke of undetermined source” to include atrial mechanical dysfunction as a causal substrate. Importantly, the predictive value of LASr goes beyond the CHA_2_DS_2_-VASc score, reinforcing its utility in more refined risk stratification [71]. As such, LASr may ultimately complement conventional risk scores in identifying patients who could benefit from closer monitoring or early anticoagulation strategies.

The scope of LASr reaches further still, touching systemic diseases that influence the heart indirectly. In hypertension, the most common cardiovascular risk factor worldwide, progressive ventricular stiffening secondarily increases atrial afterload. Even before enlargement occurs, LASr is reduced, flagging subclinical myocardial stiffening and predicting progression to overt heart failure [72]. In diabetes mellitus, LASr reductions correlate with early diastolic dysfunction and serve as a sensitive marker of myocardial involvement before overt symptoms appear [73]. In obesity, chronic volume overload, neurohormonal activation, and adipose infiltration depress LASr and may help explain the heightened risk of AF and HF in this population [74]. Collectively, these findings establish LASr as a marker of systemic cardiometabolic stress and an early indicator of atrial cardiomyopathy in high-risk patients.

In infiltrative cardiomyopathies, LASr has both diagnostic and prognostic relevance. In cardiac amyloidosis, reservoir strain falls dramatically, reflecting the inability of the infiltrated atrial wall to expand [75]. This impairment often precedes volumetric enlargement, and the degree of LASr reduction predicts arrhythmic events and adverse outcomes. In hypertrophic cardiomyopathy (HCM), reduced LASr correlates with the severity of diastolic dysfunction and predicts arrhythmic risk [76]. Similarly, in Fabry disease, LASr reduction has been described even in early stages, reinforcing its potential as a biomarker of subclinical cardiac involvement [77]. Across these entities, LASr offers a simple non-invasive means to identify early myocardial infiltration or fibrosis and to monitor disease progression or therapeutic response.

Another systemic condition that has drawn attention is chronic kidney disease (CKD). Patients with CKD display impaired LASr even before dialysis initiation, linking uremic cardiomyopathy to elevated arrhythmic and embolic risk [78]. LASr measurement in CKD may therefore provide an integrative index of the cardiovascular burden imposed by renal dysfunction, bridging renal and cardiac risk assessment.

Our study group extended LASr into conditions beyond the traditional cardiovascular field. In IPF, we observed reduced LASr despite preserved LVEF, suggesting an early atrial–ventricular–pulmonary interaction [23]. In pregnancy we demonstrated that reduced LASr in women with gestational hypertension predicted persistent hypertension postpartum [21, 22]. These investigations highlight the translational versatility of LASr, supporting its role as a cross-disciplinary marker of cardiovascular adaptation and systemic stress.

Collectively LASr provides a unifying framework that links valvular, vascular, and systemic disease through the lens of atrial mechanics—offering diagnostic, prognostic, and therapeutic insights that extend well beyond traditional imaging parameters.

Table 3 (Ref. [21, 22, 23, 24, 25, 26, 66, 67, 68, 69, 70, 72, 73, 74, 75, 76, 77, 78]) summarizes LASr assessment by 2D-STE analysis in valvular disease, stroke, and systemic disorders.

**Table 3.** **2D-STE–derived LASr assessment in valvular disease, stroke, and systemic disorders**.

Condition	LASr finding	Clinical implication
Aortic stenosis [26, 66]	Reduced LASr (<23–25%)	Identifies high-risk moderate AS, predicts events
Post-TAVI [67]	Improvement in LASr	Predicts symptomatic recovery
Mitral regurgitation [68]	Preserved vs reduced LASr	Distinguishes compensated vs decompensated MR
Mitral stenosis [69]	Lower LASr correlates with severity	Reflects hemodynamic burden
Ischemic stroke without AF [70]	Reduced LASr	Supports atrial cardiomyopathy concept
AF with LAA thrombus [24, 25]	Reduced LASr (<10–12%)	Predicts thrombus/SEC
Hypertension [72]	Reduced LASr	Detects early diastolic dysfunction
Diabetes [73]	Reduced LASr	Early myocardial dysfunction
Obesity [74]	Reduced LASr	Explains elevated AF/HF risk
Amyloidosis [75]	Severely reduced LASr	Diagnostic and prognostic marker
Hypertrophic cardiomyopathy [76]	Reduced LASr	Predicts arrhythmic risk
Fabry disease [77]	Reduced LASr	Early marker of cardiac involvement
CKD [78]	Reduced LASr	Links uremic cardiomyopathy to embolic risk
IPF [23]	Reduced LASr	Early LV diastolic dysfunction
Gestational hypertension [21, 22]	Reduced LASr	Predicts persistent postpartum hypertension

Note: 2D, two-dimensional; AF, atrial fibrillation; AS, aortic stenosis; CKD, chronic kidney disease; HF, heart failure; IPF, idiopathic pulmonary fibrosis; LAA, left atrial appendage; LASr, left atrial reservoir strain; LV, left ventricular; MR, mitral regurgitation; SEC, spontaneous echo contrast; STE, speckle tracking echocardiography; TAVI, transcatheter aortic valve implantation.

## 5. Integrating LASr Into a Practical Clinical Workflow

**Fig. 3.**
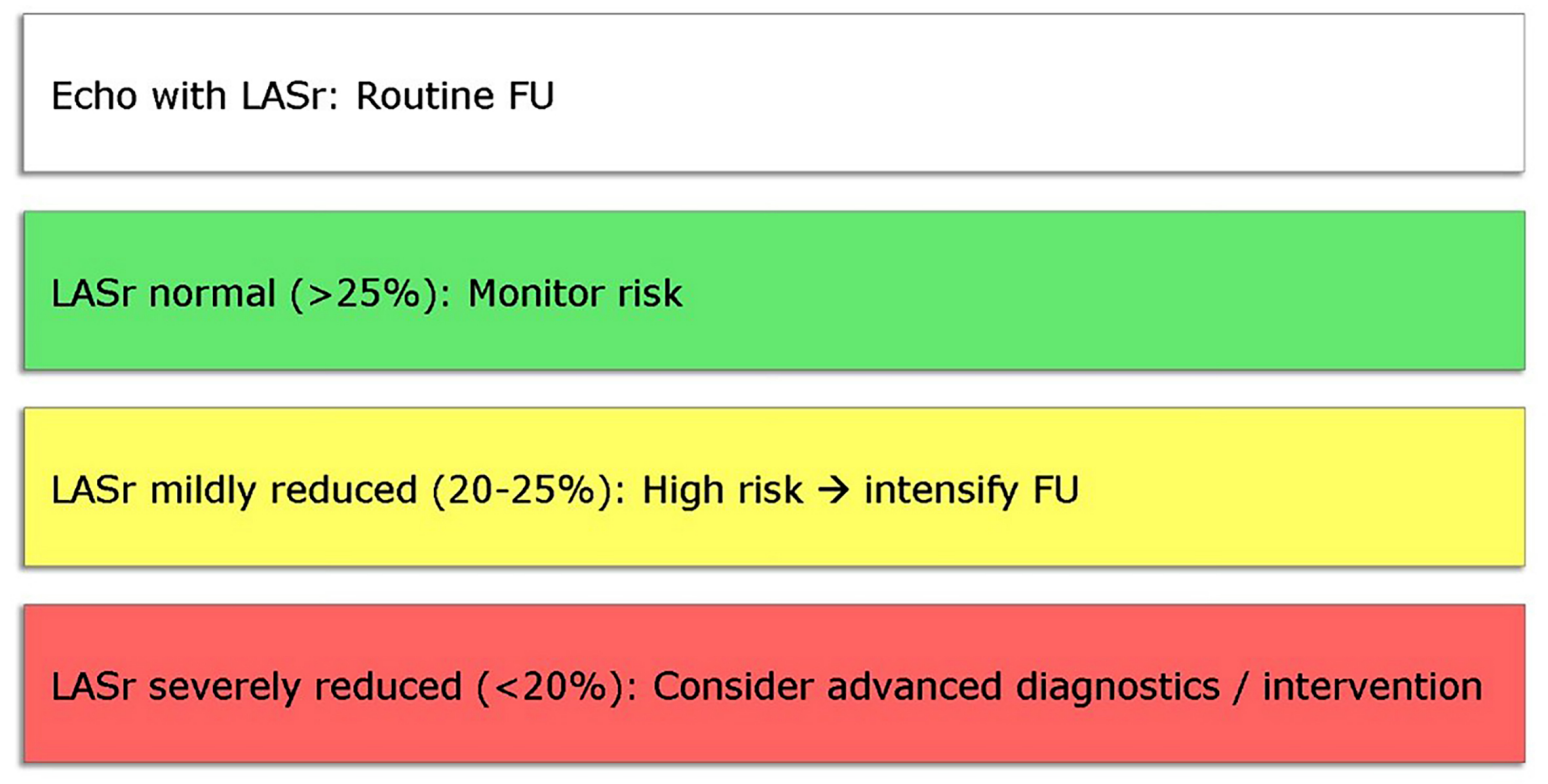
**Proposed echocardiographic LASr-guided clinical workflow**. LASr, left atrial reservoir strain; FU, follow-up.

A clinically meaningful approach to LASr involves embedding this parameter into structured workflows for patient evaluation, risk stratification, and follow-up (Fig. 3).

The overarching aim is to move beyond LASr as an isolated imaging metric and establish it as a dynamic, decision-support biomarker applicable across multiple stages of cardiovascular care.

The proposed LASr-guided workflow begins with screening and baseline assessment. In patients presenting with dyspnea, suspected heart failure, AF, or valvular disease, LASr should be measured alongside conventional echocardiographic indices. A normal LASr (>25%) typically indicates preserved atrial compliance and low near-term cardiovascular risk. These individuals can be reassured and monitored with routine follow-up, similar to patients with normal LVEF who require no additional intervention.

When LASr is mildly reduced (20–25%), it signals incipient atrial dysfunction and increased risk of progression. In this group, clinicians should intensify surveillance, optimize modifiable risk factors (hypertension, diabetes, obesity), and consider complementary investigations such as natriuretic peptides or ambulatory rhythm monitoring. In AF, a mildly reduced LASr may help estimate recurrence risk after cardioversion or ablation, while in valvular disease it can support earlier intervention before overt enlargement or symptoms develop.

Patients with severely reduced LASr (<20%) require proactive diagnostic and therapeutic management. At this threshold, atrial myopathy is usually advanced, and the likelihood of adverse events—thromboembolism, persistent AF, or HF hospitalization—rises sharply. These patients warrant comprehensive evaluation, including TEE, CMR, or invasive hemodynamic studies when appropriate. In AF, LASr below ~20% may refine anticoagulation decisions beyond CHA_2_DS_2_-VASc scoring, while in valvular disease it can justify earlier surgical or transcatheter intervention. In systemic conditions, severely reduced LASr should prompt specialist cardiology referral for integrated management.

Follow-up is a key component of the LASr-guided approach. Because LASr is dynamic and therapy-responsive, repeat measurements after interventions such as TAVI, AF ablation, or guideline-directed HF therapy can provide valuable feedback on treatment efficacy. Improvement in LASr parallels symptomatic recovery and predicts better outcomes, reinforcing its potential as both a prognostic and therapeutic biomarker.

By stratifying patients into normal, mildly impaired, and severely impaired LASr categories, clinicians can personalize surveillance intensity, optimize therapeutic timing, and refine follow-up strategies. In summary, a structured LASr-guided workflow represents a bridge between advanced imaging physiology and pragmatic, precision-based cardiovascular care.

## 6. Limitations, Future Perspectives, and Clinical Integration 

No biomarker, however promising, is without caveats, and LASr is no exception. Appreciating its limitations is essential not only for accurate interpretation but also for guiding research toward solutions that will consolidate its place in clinical practice.

One of the most important and frequently cited limitations is vendor variability. Although consensus recommendations have made progress, differences remain between software packages and imaging platforms. Two laboratories imaging the same patient under identical conditions may report slightly different LASr values, typically differing by two to four percentage points [37]. This degree of variation becomes clinically relevant when decision-making hinges on narrow diagnostic thresholds such as 20% or 23%. For now, the most pragmatic approach is to interpret LASr trends within the same patient using a consistent platform, while exercising caution when comparing values across vendors. Whenever possible, serial follow-up should be performed on the same ultrasound system and software version to ensure longitudinal comparability. The same principle applies to different imaging modalities such as MR and CT, where distinct cut-offs for normality must also be considered. Active standardization initiatives by the EACVI/ASE/Industry Task Force, together with emerging artificial intelligence (AI)–based normalization algorithms, represent critical steps toward vendor-independent and reproducible measurements.

A second limitation is load-dependence. LASr reflects not only the structural properties of the atrial wall but also the hemodynamic conditions of the moment. Sudden reductions in preload—whether from diuresis, acute dehydration, or positional maneuvers—can lower LASr values. Controlled studies in healthy volunteers have demonstrated these effects, showing that LASr falls with reduced venous return, although the proportional change is smaller than that of atrial volumes [46]. Clinicians should therefore always interpret LASr within the broader hemodynamic and pharmacological context, considering acute shifts in volume status, blood pressure, or vasoactive therapy that may transiently influence results.

Heart rhythm effects represent another key challenge. In sinus rhythm, reservoir, conduit, and booster phases can be clearly separated. In AF, however, the loss of organized atrial contraction eliminates the booster component, while reservoir strain varies from beat to beat depending on cycle length. Despite this, reservoir strain remains reproducible if multiple beats are averaged, and several studies have shown that it retains strong prognostic value even in AF [79, 80]. To enhance accuracy, averaging at least three consecutive beats in AF and five in sinus rhythm is recommended, along with ECG gating and stable image acquisition. Still, careful acquisition is needed, and clinicians must recognize the limitations of single-beat measurements in irregular rhythms.

Feasibility is generally high, with more than 90% of patients providing analyzable images in routine practice. Yet certain populations—those with obesity, chronic lung disease, or postsurgical anatomy—pose difficulties. In these cases, multimodality imaging becomes valuable. CMR offers high spatial resolution and direct correlation with fibrosis [2, 48], while CT can provide strain analysis when images are already being acquired for other indications [40, 41, 42]. Importantly, the ability to reproduce LASr across modalities confirms that it represents a true physiological signal rather than a technique-specific artifact. These complementary approaches underscore that LASr is not tied to a single modality but is a physiological measure reproducible across technologies.

Another interpretative limitation arises from extracardiac and anatomical factors. LASr magnitude may be reduced in healthy individuals with various degrees of anterior chest wall deformity or pectus excavatum, even in the absence of intrinsic myocardial dysfunction [81]. Such findings emphasize the need to consider patient morphology and imaging windows when interpreting abnormally low values.

Finally, the lack of universal cut-off values remains a fundamental challenge. Despite multiple studies proposing disease-specific thresholds, inter-vendor differences, population heterogeneity, and load-dependence prevent the establishment of universally accepted reference limits. Until standardized normative data become available, LASr should be interpreted as a continuous variable—where relative changes and trends over time may be more informative than absolute numbers.

Looking ahead, several future perspectives hold promise for enhancing the role of LASr in clinical practice.

Artificial intelligence represents the most transformative opportunity. AI-driven analysis can automate border detection, reduce observer dependence, and harmonize values across vendors. Pilot studies have shown that deep learning models can reproduce strain measurements with accuracy comparable to expert readers, and efforts are under way to validate AI-assisted LASr in large, multicenter cohorts [82, 83]. Beyond reproducibility, AI may enable the development of integrated risk models that combine LASr with clinical variables, biomarkers, and imaging features to provide individualized prognostication. In the near future, AI integration could facilitate real-time LASr reporting directly within echocardiographic workflows.

Another emerging concept is that of atrial cardiomyopathy. Traditionally, atrial dysfunction was regarded as secondary to ventricular disease or arrhythmia. Increasingly, it is recognized as a disease entity in its own right, encompassing structural remodeling, electrical instability, and a prothrombotic milieu. LASr may serve as the principal imaging biomarker of this entity, providing a quantifiable, reproducible index that integrates the mechanical and structural dimensions of atrial health. This shift could lead to refined definitions of atrial cardiomyopathy, with LASr thresholds eventually serving as diagnostic criteria, much as ejection fraction defines systolic heart failure.

Preventive cardiology represents another exciting frontier. Because LASr declines early—often before symptoms or chamber enlargement—it could serve as a screening tool in at-risk populations such as hypertensive or diabetic patients, individuals with obesity, or cancer survivors exposed to cardiotoxic chemotherapy. Detecting atrial dysfunction at this subclinical stage might enable interventions to prevent progression to overt AF or heart failure. However, it must also be considered that studies consistently demonstrate a wide overlap of standard deviations when comparing different groups, with mean values that, although statistically distinct, remain close in absolute terms. This overlap limits the diagnostic accuracy of single LASr values in individual patients, reinforcing the need for serial assessment and longitudinal follow-up. Long-term studies are required to determine whether LASr-guided prevention translates into improved outcomes.

Integration into clinical practice and future guidelines will be the next milestone. For this to happen, cut-offs must be standardized, acquisition protocols unified, and cost-effectiveness demonstrated. Registries and prospective multicenter trials will be critical. Already, some centers have begun incorporating LASr into routine echocardiography reports, particularly for patients with dyspnea of unclear origin, AF, or valvular disease. Training programs and standardized reporting templates will be essential for widespread adoption. Unlike static morphologic measures, LASr is dynamic—it responds to interventions and therapy. It improves after TAVI in aortic stenosis, after ablation in AF, and after optimized medical therapy in heart failure. Monitoring these changes may allow clinicians to track treatment efficacy and guide individualized management in real time.

## 7. Conclusions

Left atrial reservoir strain is a sensitive marker of atrial health, integrating atrial compliance and ventricular diastolic properties. It detects dysfunction earlier than conventional parameters and provides consistent diagnostic and prognostic value across heart failure, atrial fibrillation, valvular disease, stroke, and systemic disorders. Despite limitations—vendor variability, load dependence, and rhythm effects—advances in standardization, multimodality imaging, and artificial intelligence are enhancing reliability and clinical utility. LASr is evolving from a research parameter to a practical biomarker that reflects global cardiac–atrial interaction and response to therapy. As reproducibility improves, LASr may become a routine component of cardiovascular evaluation, offering a dynamic, physiology-based measure that refines risk stratification and enables earlier detection of disease progression.

## Availability of Data and Materials

Data extracted from included studies are deposited to Zenodo (https://zenodo.org) and will be publicly available as of the date of publication.
